# The Phenotypic and Mutational Spectrum of the FHONDA Syndrome and Oculocutaneous Albinism: Similarities and Differences

**DOI:** 10.1167/iovs.63.1.19

**Published:** 2022-01-14

**Authors:** Charlotte C. Kruijt, Libe Gradstein, Arthur A. Bergen, Ralph J. Florijn, Benoit Arveiler, Eulalie Lasseaux, Xavier Zanlonghi, Laura Bagdonaite-Bejarano, Anne B. Fulton, Claudia Yahalom, Anat Blumenfeld, Yonatan Perez, Ohad S. Birk, Gerard C. de Wit, Nicoline E. Schalij-Delfos, Maria M. van Genderen

**Affiliations:** 1 Bartiméus Diagnostic Center for Complex Visual Disorders, Zeist, The Netherlands; 2 Department of Ophthalmology, Leiden University Medical Center, Leiden, The Netherlands; 3 Department of Ophthalmology, Soroka Medical Center and Clalit Health Services, Faculty of Health Sciences, Ben Gurion University of the Negev, Beer Sheva, Israel; 4 Department of Human Genetics, Amsterdam University Medical Center, Location AMC, Amsterdam, The Netherlands; 5 The Netherlands Institute for Neurosciences (NIN-KNAW), Amsterdam, The Netherlands; 6 Department of Ophthalmology, Academic Medical Center, Amsterdam, The Netherlands; 7 Maladies Rares: Génétique et Métabolisme (MRGM), Inserm U1211, University of Bordeaux, Bordeaux, France; 8 Department of Medical Genetics, CHU Bordeaux, Bordeaux, France; 9 Centre de Compétence Maladie Rares, Clinique Pluridisciplinaire Jules Verne, Nantes, France; 10 Department of Ophthalmology, Boston Children's Hospital, Boston, Massachusetts, United States; 11 Department of Ophthalmology, Harvard Medical School, Boston, Massachusetts, United States; 12 Faculty of Medicine, Hebrew University of Jerusalem, Israel; Department of Ophthalmology, Hadassah Medical Center, Jerusalem, Israel; 13 The Morris Kahn Laboratory of Human Genetics, National Institute for Biotechnology in the Negev and Faculty of Health Sciences, Ben Gurion University of the Negev, Beer Sheva, Israel; 14 Genetics Institute, Soroka Medical Center, Ben Gurion University of the Negev, Beer Sheva, Israel; 15 Department of Ophthalmology, University Medical Center Utrecht, Utrecht, The Netherlands

**Keywords:** SLC38A8, FHONDA, foveal hypoplasia, misrouting, melanin

## Abstract

**Purpose:**

The purpose of this study was to further expand the mutational spectrum of the Foveal Hypoplasia, Optic Nerve Decussation defect, and Anterior segment abnormalities (FHONDA syndrome), to describe the phenotypic spectrum, and to compare it to albinism.

**Subjects and Methods:**

We retrospectively collected molecular, ophthalmic, and electrophysiological data of 28 patients molecularly confirmed with FHONDA from the Netherlands (9), Israel (13), France (2), and the United States of America (4). We compared the data to that of 133 Dutch patients with the 3 most common types of albinism in the Netherlands: oculocutaneous albinism type 1 (49), type 2 (41), and ocular albinism (43).

**Results:**

Patients with FHONDA had a total of 15 different mutations in *SLC38A8*, of which 6 were novel. Excluding missing data, all patients had moderate to severe visual impairment (median visual acuity [VA] = 0.7 logMAR, interquartile range [IQR] = 0.6–0.8), nystagmus (28/28), and grade 4 foveal hypoplasia (17/17). Misrouting was present in all nine tested patients. None of the patients had any signs of hypopigmentation of skin and hair. VA in albinism was better (median = 0.5 logMAR, IQR = 0.3–0.7, *P* 0.006) and the phenotypes were more variable: 14 of 132 without nystagmus, foveal hypoplasia grades 1 to 4, and misrouting absent in 16 of 74.

**Conclusions:**

Compared to albinism, the FHONDA syndrome appears to have a more narrow phenotypic spectrum, consisting of nonprogressive moderately to severely reduced VA, nystagmus, severe foveal hypoplasia, and misrouting. The co-occurrence of nystagmus, foveal hypoplasia, and misrouting in the absence of hypopigmentation implies that these abnormalities are not caused by lack of melanin, which has important implications for understanding the pathogenesis of these features.

Until 2006, misrouting of the visual pathways was only described in albinism and believed to be secondary to the lack of ocular pigmentation.^1^^–^^4^ In 2006, van Genderen et al. first reported the combination of misrouting and foveal hypoplasia in patients without albinism.^5^ The patients described in this paper showed similarities to a family described by Pal et al. with foveal hypoplasia and anterior segment dysgenesis.^6^ In 2013, the term FHONDA was introduced for the disorder, which is short for Foveal Hypoplasia, Optic Nerve Decussation defects and Anterior segment dysgenesis.^7^ Poulter et al. discovered that mutations in the *SLC38A8* gene were responsible for the FHONDA syndrome and demonstrated that in embryonic Medaka fish, knockdown of both orthologs of *SLC38A8* did not result in any pigmentation defect of the eye or tegumen.^8^ Perez et al. described 9 patients with homozygous *SLC38A8* mutations who had a combination of foveal hypoplasia and nystagmus also without any signs of hypopigmentation.^9^

FHONDA and albinism share the clinical features of nystagmus, foveal hypoplasia, and misrouting, but because of the lack of pigmentation defect in Medaka fish, and in earlier reported patients with FHONDA it seems that albinism and FHONDA are distinct disorders.

The phenotypic spectrum of albinism has been extensively investigated and appears to be very broad.^10^^–^^15^ Albinism is a heterogeneous condition, and even patients who have mutations in the same gene also show variable clinical features. Visual acuity in albinism ranges from very poor to normal and foveal hypoplasia of all grades has been described, from absent (grade 0) to severe (grade 4). None of the clinical features is universal for all patients with albinism, with nystagmus being absent in at least 7% and misrouting in 16%.^10^ FHONDA appears to be rare, and seems more homogeneous than albinism. Since the identification of the first patient, only a few cases have been reported.^5^^–^^9^^,^^14^^,^^16^^–^^20^ The disorder is unknown to most clinicians and therefore is not adequately recognized. The purpose of this study is to further define the phenotypic and genetic spectrum of FHONDA, and to compare its presentation to albinism.

## Methods

The study was approved by the Medical Ethics Committee of Leiden University Medical Center and adhered to the tenets of the Declaration of Helsinki.

### Patients With FHONDA

We included 28 patients with FHONDA from 4 countries, 12 male patients and 17 female patients, aged 1 to 71 years (median age 24 years). All patients, or their affected siblings, had two likely disease-causing variants in *SLC38A8*, and had records on their pigmentation status and ophthalmic findings.

Patients with FHONDA from the Netherlands (9 patients and 5 families) were diagnosed at the Bartiméus Institute. Additional patients came from tertiary referral centers in Israel (13 patients, 7 families), France, (2 patients, 1 family), and the United States of America (4 patients, 3 families). Five patients from the Netherlands, nine from Israel, and one patient from France were previously reported, however, several clinical features were not described, for instance, grading of foveal hypoplasia.^5^^,^^7^^,^^8^^,^^14^ Data were obtained through medical record review for best-corrected visual acuity (VA), refractive error, pigmentation of eyes, skin, and hair, slit lamp examination, ophthalmoscopy, fundus photography, optical coherence tomography (OCT) scans, and multichannel Visual Evoked Potentials (VEPs) tests. We used a grading scheme for foveal hypoplasia previously described by Thomas et al., with grade 0 indicating normal foveal structure, grades 1 and 2 not having incursion of the inner retinal layers, and grades 3 and 4 also affecting the photoreceptor differentiation (Supplementary Table S1).^21^ Multichannel VEPs were obtained with Espion E2 or E3 (Diagnosys LLC, Cambridge, UK), according to ISCEV standards.^22^ A chiasm coefficient was calculated and the cutoff values were used from the study of Kruijt et al.^23^

### FHONDA Versus Albinism

We compared the phenotypic spectrum of FHONDA to that of 133 patients with genetically confirmed albinism, 99 male patients and 34 female patients, aged 0 to 77 years (median age 10 years). These patients had either two mutations in *TYR* (49 patients) or *OCA2* (41 patients), or a mutation in *GPR143* (43 patients). Mutations in *TYR* and *OCA2* cause oculocutaneous albinism (OCA) type 1 and 2, respectively, and those in *GPR143* cause ocular albinism (OA1), the 3 most common types of albinism in the Netherlands. We used IBM SPSS Statistics software version 22 to perform statistical analysis. Data were not normally distributed, and therefore we used nonparametric tests.

## Results

All but two of the 28 patients with FHONDA were able to cooperate with VA measurement, OCT scans for grading were available in 17 of 28 cases. VEP testing was performed in 9 of 28 patients. VA could be tested in 57 of 90 patients with albinism, in 43 of 90 patients, OCT-scans were obtained, and in 58 of 90 patients VEP tests were done.

### FHONDA (*N* = 28)

**Figure 1.**
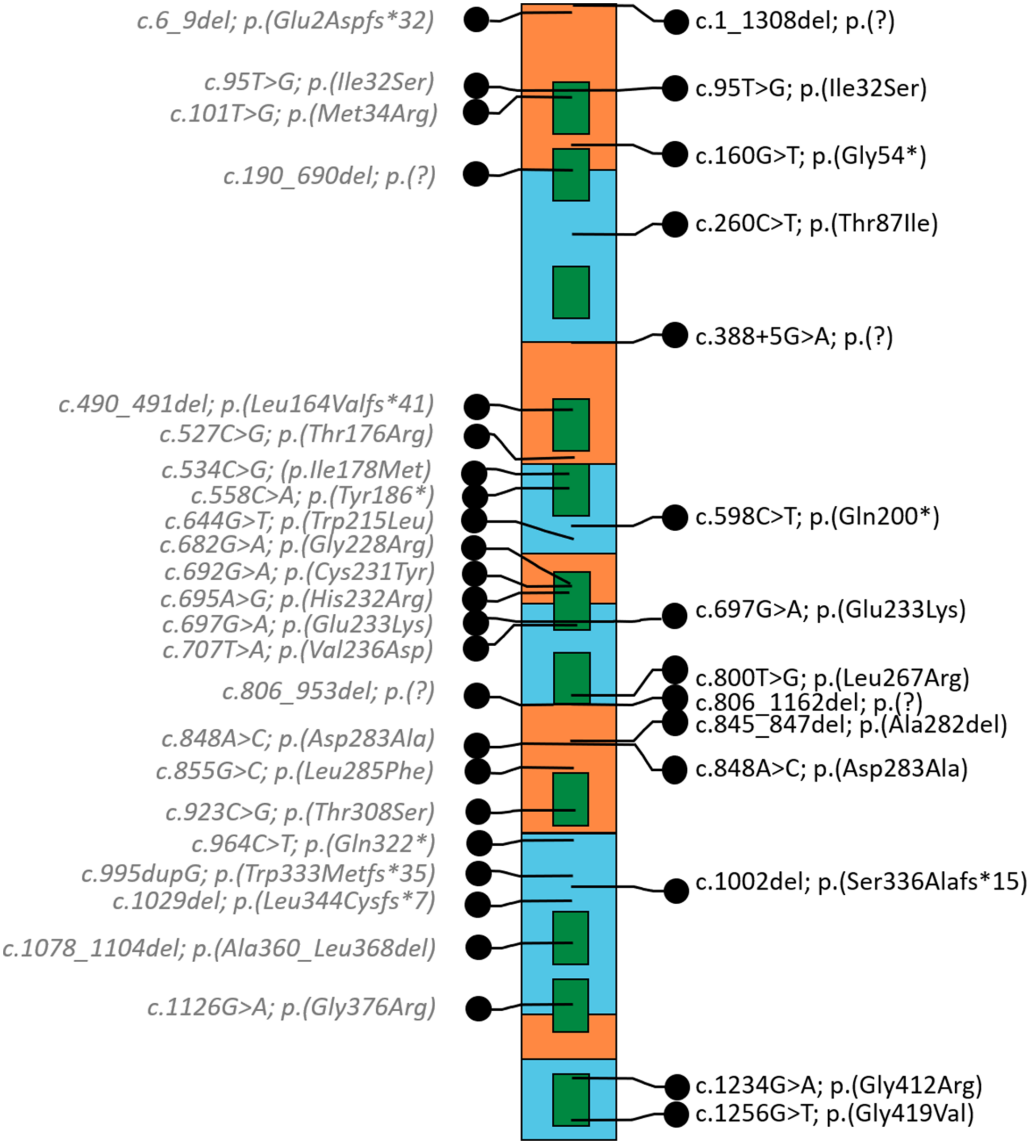
Schematic representation of the *SLC38A8* gene. The location of the variants found in this study patients is represented on the right and of those retrieved from literature on the left, in grey. Exons are illustrated in orange and blue, transmembrane domains in green. Deletions are depicted at the location of the first deletion coordinate.

Molecular analyses and demographic characteristics of the 28 patients with FHONDA are presented in Table 1. Patients had a total of 15 different mutations in *SLC38A8*, consisting of missense, (inframe) deletions, frameshift, and truncating mutations located over the entire gene (Table 1, Fig. 1). We report six novel mutations: c.260C > T; p.(Thr87Ile) and c.800T > G; p.(Leu267Arg) in the Dutch family III, c.160G > T; p.(Gly54*) and c.388 + 5G > A; p.(?) in family VII from the United States, c.(805 + 1_806–1)_(1162 + 1_1163–1)del; p.(?) (deletion exon 7 and 8) in the French family VI and family VIII from the United States, respectively, and c.1256G > T; p.(Gly419Val) in family VIII.

**Table 1.** Molecular Analyses Patients With FHONDA

Family	Descent	ID	Mutations SLC38A8 Gene
I*	*Afghani*	801	Homozygous c.1002del; p.(Ser336Alafs*15)
I*	*Afghani*	802	Homozygous c.1002del; p.(Ser336Alafs*15)
I	*Afghani*	828	Homozygous c.1002del; p.(Ser336Alafs*15)
II*	*Dutch*	803	One large deletion and c.1234G > A; p.(Gly412Arg)
III	*Dutch*	804	c.260C > T; p.(Thr87Ile) and c.800T > G; p.(Leu267Arg)
III	*Dutch*	805	c.260C > T; p.(Thr87Ile) and c.800T > G; p.(Leu267Arg)^†^
IV*	*Dutch*	806	c.598C > T; p.(Gln200*) and c.845_847del; p.(Ala282del)
IV*	*Dutch*	807	c.598C > T; p.(Gln200*) and c.845_847del; p.(Ala282del)
V	*Dutch*	808	Homozygous c.598C > T; p.(Gln200*)
VI	*French*	809	c.697G > A; p.(Glu233Lys) and c.(805 + 1_806–1)_(1162 + 1_1163–1)del; p.(?) deletion exon 7 and 8
VI*	*French*	810	c.697G > A; p.(Glu233Lys) and c.(805 + 1_806–1)_(1162 + 1_1163–1)del; p.(?) deletion exon 7 and 8
VII	*Irish/French-Canadian/Puerto Rican*	811	c.160G > T; p.(Gly54*) and c.388 + 5G > A; p.(?)
VIII	*Swedish/Italian/Irish/English*	812	c.(805 + 1_806–1)_(1162 + 1_1163–1)del; p.(?) deletion exon 7 and 8 and c.1256G > T p.(Gly419Val)
IX	*Ashkenazi-Jewish*	813	Homozygous c.848A > C; p.(Asp283Ala)
IX	*Ashkenazi-Jewish*	814	Homozygous c.848A > C; p.(Asp283Ala)
X*	*Indian Jewish*	815	Homozygous c.95T > G; p.(Ile32Ser)
X*	*Indian Jewish*	816	Homozygous c.95T > G; p.(Ile32Ser)
X*	*Indian Jewish*	817	Homozygous c.95T > G; p.(Ile32Ser)
XI*	*Indian Jewish*	818	Homozygous c.95T > G; p.(Ile32Ser)
XI*	*Indian Jewish*	819	Homozygous c.95T > G; p.(Ile32Ser)
XII*	*Indian Jewish*	820	Homozygous c.95T > G; p.(Ile32Ser)
XII*	*Indian Jewish*	821	Homozygous c.95T > G; p.(Ile32Ser)
XII*	*Indian Jewish*	822	Homozygous c.95T > G; p.(Ile32Ser)
XII*	*Indian Jewish*	823	Homozygous c.95T > G; p.(Ile32Ser)
XIII	*Jewish;* *Ashkenazi-Lebanon-Syria/Yemenite-Afghanistan*	824	c.848A > C; p.(Asp283Ala) and whole gene deletion
XIV	*Indian Jewish*	825	Homozygous c.95T > G; p.(Ile32Ser)
XIV	*Indian Jewish*	826	Homozygous c.95T > G; p.(Ile32Ser)
XV	*Ashkenazi-Jewish*	827	Homozygous c.848A > C; p.(Asp283Ala)

* Families are partially described previously, but we obtained additional information.^5^^,^^7^^–^^9^^,^^14^ ^†^Molecular analysis in siblings.

About two thirds of the patients (i.e. 18/28 patients from 8 families, were of south-western Asian origin, Afghani, and Indian, and Ashkenazi Jewish descent). All had homozygous mutations, except the patient from family XIII. The 11 patients from the 4 Indian Jewish families were all homozygous for c.95T > G; p.(Ile32Ser). The c.848A > C variant was found in all Ashkenazi-Jewish patients. The patient from family XIII was of a mixed origin, includingAshkenazi, Lebanon-Syria, and Yemenite-Afghanisti descent, and had a compound heterozygous mutation c.848A > C; p.(Asp283Ala), and a whole gene deletion. In contrast, 9 of 10 (partially) western European patients were compound heterozygotes.

**Figure 2.**
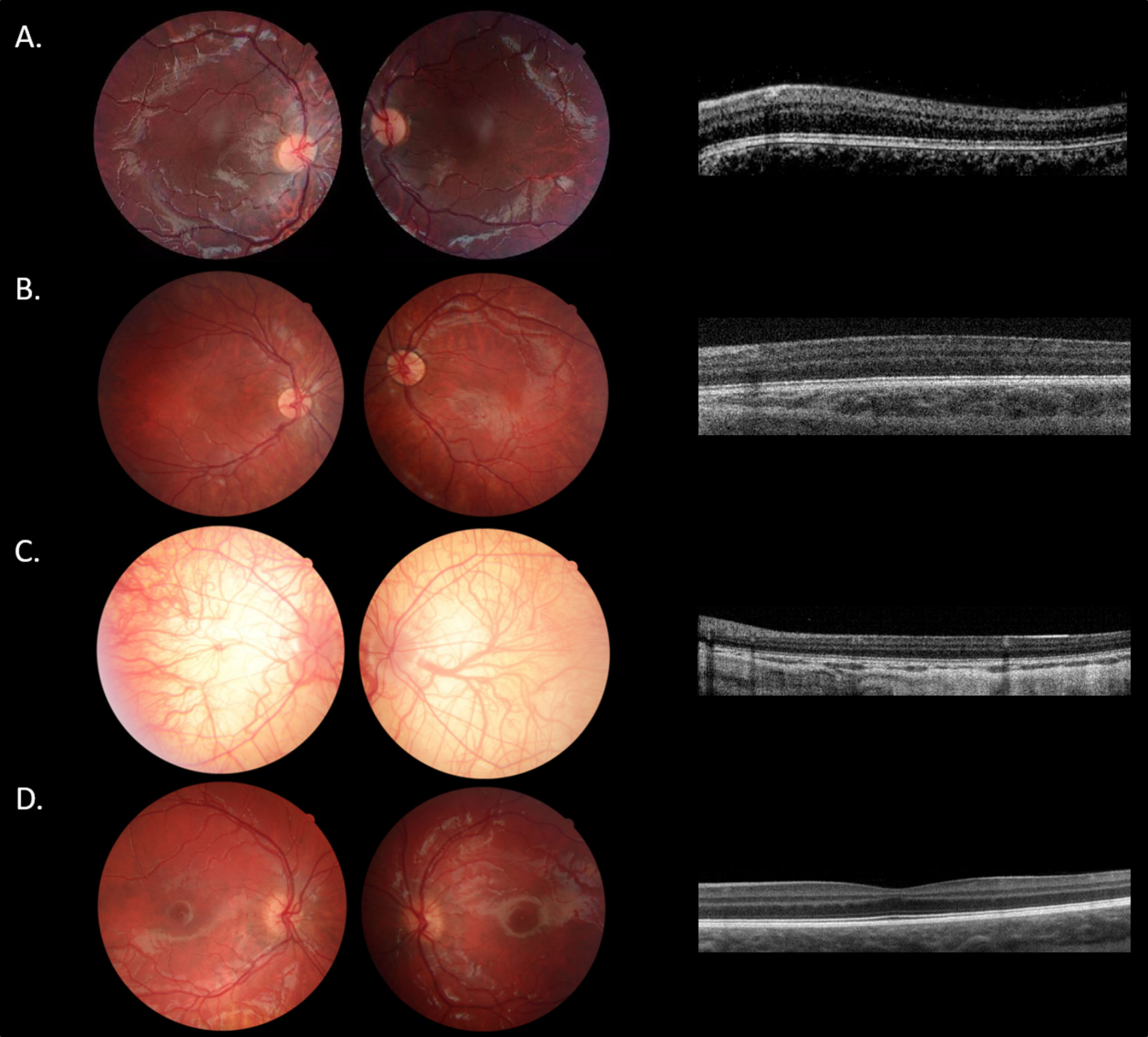
Clinical results. (**A**) Fundus images and optical coherence tomography scans of the clinical study patient VIII-812. (**B**) Fundus image and optical coherence tomography scan of clinical study patient III-804. (**C**) Example of a severely affected albinism patient. Note the completely translucent fundus accompanied by grade four foveal hypoplasia.^21^ The patient had two mutations in the TYR gene (c.164G > A and c.896G > A), visual acuity was 1.0 logMAR, and patient had nystagmus, and complete iris translucency. (**D**) Example of an albinism patient without obvious hypopigmentation of the fundus, comparable to patient FIII-804 with FHONDA. Note the minimal foveal hypoplasia grade 1.^21^ Other manifestations were also mild, with a suboptimal visual acuity of 0.3 logMAR, nystagmus, and absence of iris translucency. The patient was homozygote for mutation c.1037-7T > A in the TYR gene. All optical coherence tomography scans of the left eye and right eye had the same grade of foveal hypoplasia.

**Figure 3.**
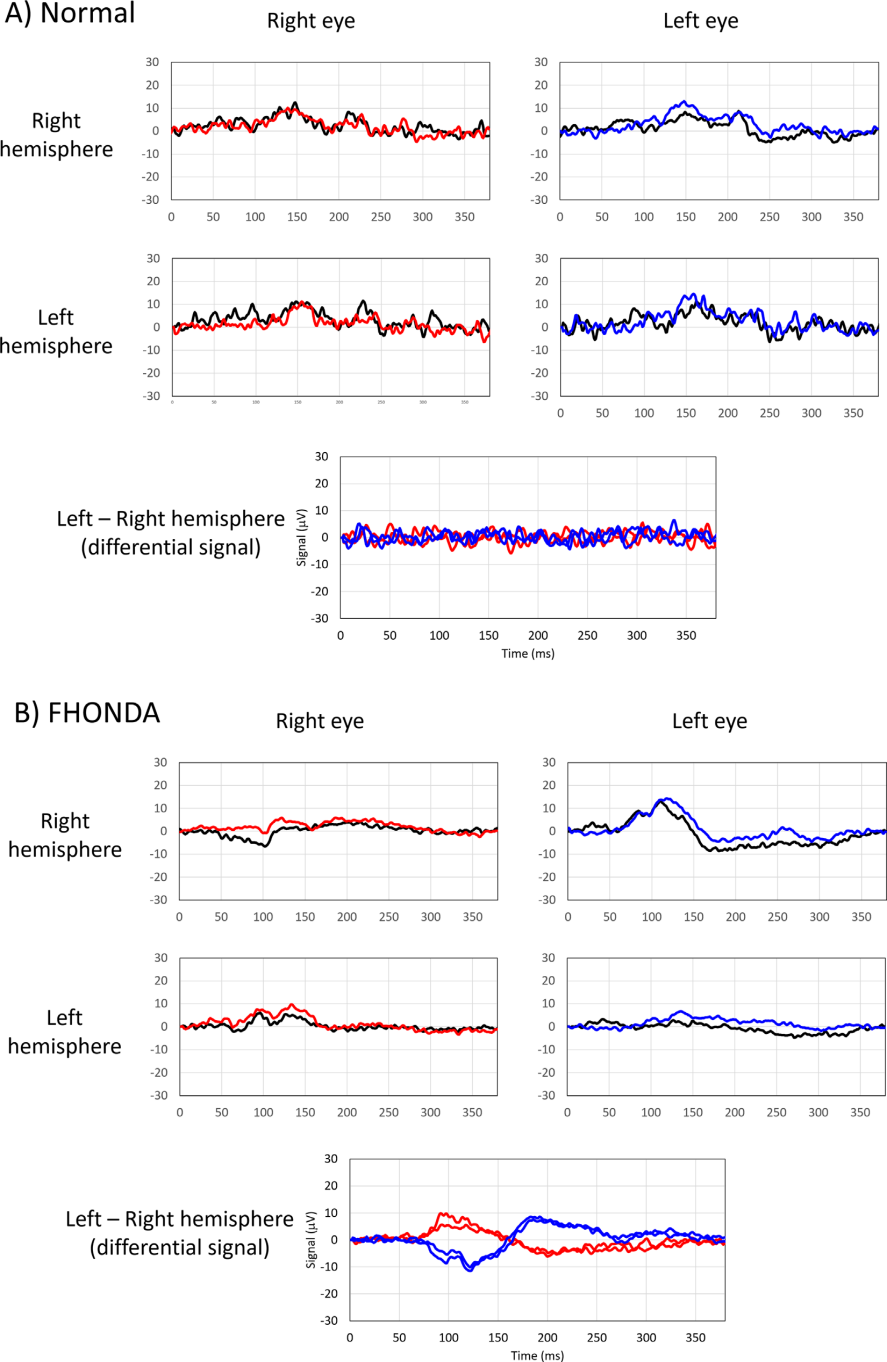
Chiasmal misrouting in FHONDA. (**A**) Visually evoked pattern onset potentials recordings of a normal subject. Note correlation of responses recorded from right and left eye in the differential signal. (**B**) Visually evoked pattern onset potentials from clinical study patient V-808. Note the asymmetry in the differential signal recorded from right eye and left eye. The chiasm coefficient was –0.9, indicating chiasmal misrouting.^23^^–^^25^

Phenotypic data of patients with FHONDA are presented in Table 2. Patients (25/28) had a median VA of 0.7 logMAR (interquartile range [IQR] = 0.6-0.8). None of the patients was emmetropic, with moderate (1 to 2 diopters) to severe (>2 diopters) astigmatism as the most characteristic refractive error. Only 4 of 28 patients had anterior segment abnormalities (i.e. posterior embryotoxon). All patients had nystagmus (28/28), grade four foveal hypoplasia (17/17), and misrouting of the optic nerve fibers at the chiasm (9/9). All (28/28) patients had normal pigmentation of skin, and/or hair compared to family members and no iris translucency. Fundus imaging showed a lightly pigmented midperiphery in an infant of 6 months of age (VII-811), but some pigmentation was already seen in the macular region. Figures 2A and 2B show examples of foveal hypoplasia and fundus images in patients with FHONDA and patients with albinism. Figure 3 shows misrouting in a patient with FHONDA.

**Table 2.** Phenotype Patients With FHONDA

Family ID	Refraction RE, LE^†^	VA^‡^	Nystagmus Type	Iris Translucency	Foveal Hypoplasia^§^	Anterior Segment	Hypopigmentation Fundus	Misrouting
I*-801	+1.25D/–1.75Dx179 +1.25D/–1.25Dx179	0.7	Horizontal jerk	No	Grade 4	Posterior embryotoxon	No	Yes
I*-802	–4.5D/–4.25Dx88 –5.75D/–4.75Dx177	0.8	Horizontal jerk	No	Grade 4	Posterior embryotoxon	No	Yes
I-828	ND	ND	Horizontal jerk	No	ND	Normal	No	Yes
II*-803	+2.0D/–2.0Dx5 +2.25D/–2.0Dx180	0.7	Horizontal jerk	No	Grade 4	Posterior embryotoxon	No	Yes
III-804	+4.5D/–2.75Dx4 +3.5D/–3.75Dx174	0.8	Horizontal jerk	No	Grade 4	Normal	No	Yes
III-805	+4.75D/–2.00x180 +4.25D/–1.50x11	0.8	Horizontal jerk	No	ND	Normal	No	Yes
IV*-806	+6.0D/–3.5Dx175 +6.5D/–4.0Dx15	0.7	Horizontal jerk	No	Grade 4	Normal	No	Yes
IV*-807	+7.0D/–2.0Dx180 +7.5D/–2.5Dx180	1.0	Horizontal jerk	No	Grade 4	Posterior embryotoxon	No	Yes
V-808	+10.25D/–1.25Dx17 +10.25D/–2.0Dx176	0.8	Horizontal jerk	No	Grade 4	Normal	No	Yes
VI-809	+7.0D/–1.0Dx180 +7.25D/–2.0Dx180	0.9	Horizontal jerk	No	Grade 4	Normal	No	ND
VI-810	+2.75D/–1.75Dx180 +3.5D/–1.75Dx20	0.7	Horizontal jerk	No	Grade 4	Normal	No	ND
VII-811	0.0D/–2.5Dx180 0.0D/–2.5Dx180	ND	Horizontal jerk	No	Grade 4	Normal	Blond fundus at six months of age	ND
VIII-812	+7.75D/–4.75Dx2 +7.75D/–4.50Dx2	0.7	Yes, type unknown	No	Grade 4	Normal	No	ND
IX-813	+4.00D/–1.00Dx166 +6.25D/–2.75Dx25	0.6	Horizontal jerk	No	Grade 4	Normal	No	ND
IX-814	–3.25D/–1.25Dx45 –3.25D/–2.00Dx170	0.6	Horizontal jerk	No	Grade 4	Normal	No	ND
X*-815	+0.25D/–3.5Dx180 +0.25D/–3.75Dx25	0.8	Horizontal jerk	No	Grade 4	Normal	No	ND
X*-816	+4.25D/–2.75Dx8 +5.0D/–3.0Dx180	0.8	Horizontal jerk	No	Grade 4	Normal	No	ND
X*-817	+4.0D/–3.5Dx180 +4.25D/–3.25Dx170	0.7	Horizontal jerk	No	ND	Normal	No	ND
XI*-818	+5.5D/–2.5Dx5 +5.75D/–2.5Dx180	0.5	Periodic alternating	No	ND	Normal	No	ND
XI*-819	+6.5D/–1.75Dx180 +6.25D/–0.5Dx180	0.7	Horizontal jerk	No	Grade 4	Normal	No	ND
XII*-820	–0.5D/–1.25Dx3 –0.75D/–1.5Dx7	1.0	Horizontal jerk	No	ND	Normal	No	ND
XII*-821	+2.75D/–2.0Dx25 +3.75D/–1.5Dx160	0.6	Horizontal jerk	No	ND	Normal	No	ND
XII*-822	–11.75D/–2.0Dx14 –7.75D/–0.75Dx153	0.6	Horizontal jerk	No	ND	Normal	No	ND
XII*-823	+1.5D/–1.75Dx170 +1.0D/–1.75Dx10	0.4	Periodic alternating	No	ND	Normal	No	ND
XIII-824	–6.5D/–3.25Dx180 –6.5D/–3.25Dx180	ND	Yes, type unknown	No	ND	Normal	No	ND
XIV-825	ND	0.7	Yes, type unknown	No	ND	Normal	No	ND
XIV-826	+1.0D/–1.0Dx180 +2.0D/–0.5Dx180	0.5	Yes, type unknown	No	Grade 4	Normal	No	ND
XV-827	+3.0D/–3.0Dx10 +4.0D/–2.5Dx10	0.8	Yes, type unknown	No	ND	Normal	No	ND

ND = not determined.

* Families were partially described previously.^5^^,^^7^^–^^9^^,^^14^

† Refraction right eye (RE) and left eye (LE).

‡ Visual acuity in logMAR. VII-811 was not determined because patient was one year of age.

§ According to the grading of Thomas et al*.*^21^

### FHONDA (*N* = 28) Versus Albinism (*N* = 133)

**Figure 4.**
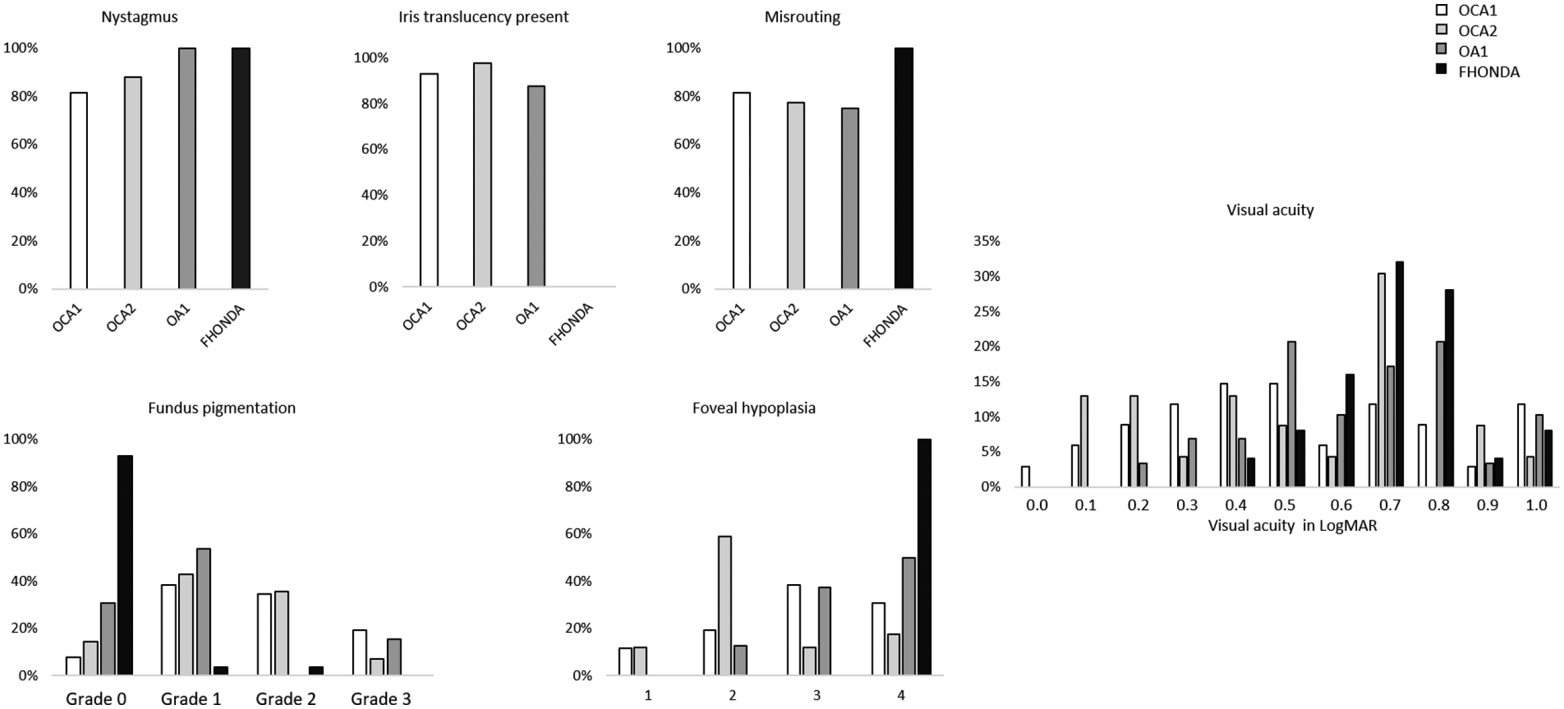
Phenotypic spectrum of FHONDA versus oculocutaneous albinism type 1, type 2, and ocular albinism. For the grading of foveal hypoplasia we used a grading scheme according to Thomas et al., with grades 1 and 2 not having incursion of the inner retinal layers, and grades 3 and 4 also affecting the photoreceptor differentiation (see Supplementary Table S1).^21^ Misrouting was determined by using cutoff values from the study of Kruijt et al. for the calculated chiasm coefficients from the multichannel visually evoked potential recordings.^23^

**Figure 5.**
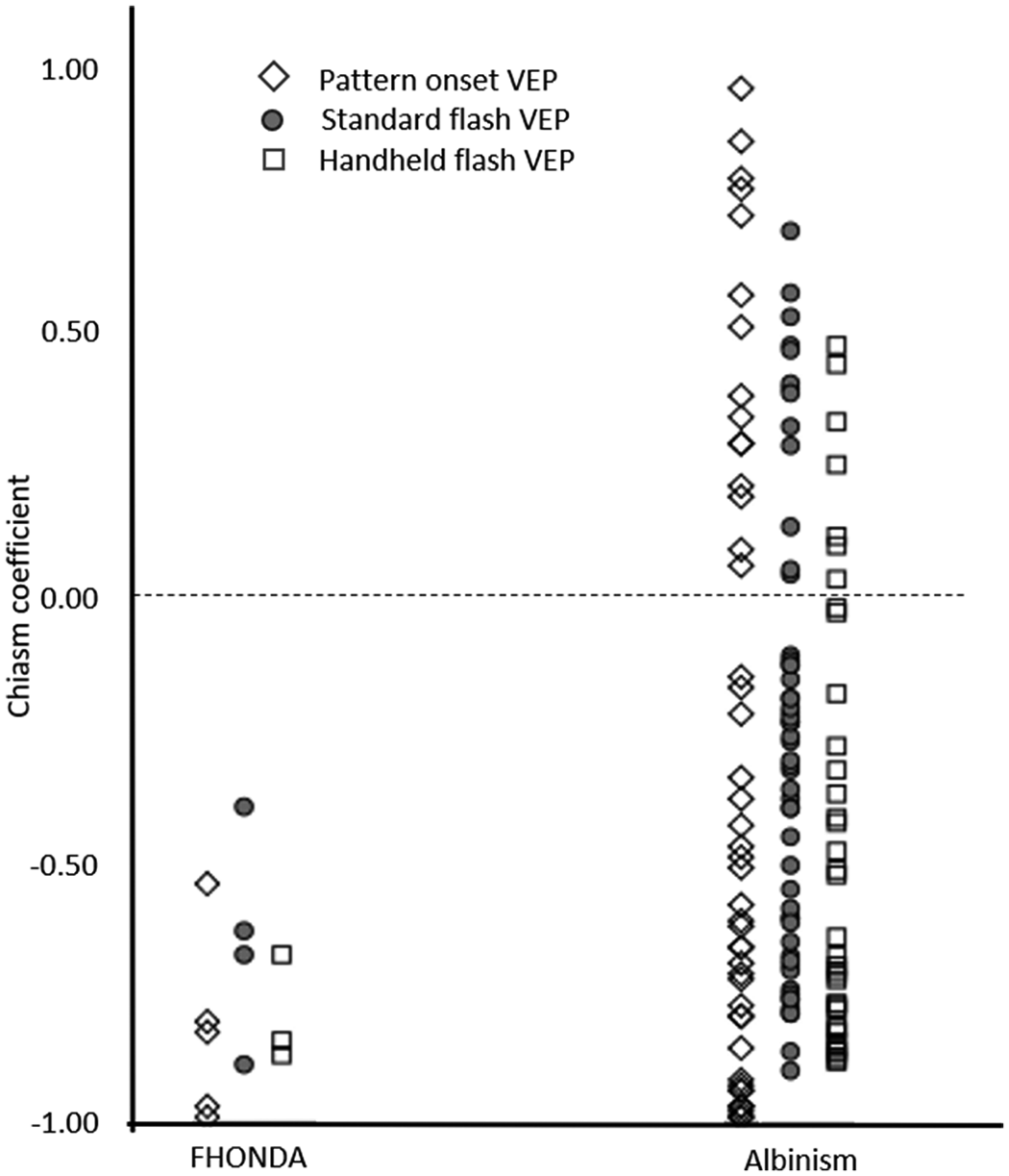
Visually evoked potentials in FHONDA and albinism. The chiasm coefficients were calculated according to Kruijt et al.^23^ A negative chiasm coefficient indicates misrouting. This figure shows all test results, independently of age. Not all patients were tested with all stimuli, sometimes only the stimulus recommended for the age was used. Note the variability in albinism and the obvious misrouting in patients with FHONDA for all stimuli.

VA was poorer and less variable in FHONDA (median = 0.7 logMAR, IQR = 0.6–0.8) compared to the 133 patients with albinism (median = 0.5 logMAR, IQR = 0.3–0.7, Mann-Whitney *U* test, *P* = 0.006). Analysis of VA of FHONDA compared to the different subtypes of albinism showed a significantly better VA in OCA1 and OCA2 (median = 0.5 logMAR, IQR = 0.3–0.7, *P* *=* 0.004, and median = 0.5 logMAR, IQR = 0.2–0.7, *P* = 0.006), respectively). Median VA in OA1 was 0.6 logMAR, IQR = 0.5 to 0.8 and was not significantly better than in FHONDA (*P* = 0.11). Besides VA, all phenotypic characteristics in OCA1, OCA2, and OA1 were more variable than in FHONDA in this study: 11% (14/132) of patients with albinism did not have nystagmus (0% in FHONDA), foveal hypoplasia varied from grades 1 to 4 (only grade 4 in FHONDA), and misrouting was absent in 22% (16/74, 0% in FHONDA; see also Fig. 4) Misrouting was more evident in FHONDA, and was detected with all stimulus types (i.e. pattern onset and flash VEP), regardless of age. In albinism, VEP recordings were much more variable, and when misrouting was confirmed by the method recommended for that age, it was not always present in recordings with other stimuli (Fig. 5).

Patients with OA1 resemble patients with FHONDA the most, because they have no hypopigmentation of the skin or hair. In addition, they have on average poorer VA and more severe foveal hypoplasia than patients with OCA in our cohort. However, almost all patients with OA1 have ocular hypopigmentation, in contrast to none of the patients with FHONDA. In our cohort, only 3 of 43 patients with OA1 were described with normal ocular pigmentation (no iris translucency AND normal fundus pigmentation). The first patient had grade one hypopigmentation of the posterior pole, according to the scheme of Kruijt et al.,^10^ which was considered normal in this fair skinned Caucasian patient. However, he had obvious hypopigmentation of the (mid)periphery with choroidal vessels clearly visible through the translucent retinal pigment epithelium. He had VA of 0.5 logMAR, nystagmus, foveal hypoplasia grade 3, and misrouting. The second patient with OA1, from Somalian descent, had high myopia, VA of 1.0 logMAR, nystagmus, and no misrouting. An OCT scan was not obtained. There was possible hypopigmentation of the fundus, but this could also be secondary to the high myopia (S-16/ S-14). The last patient, of mixed Dutch-Indonesian descent, had VA of 0.25 logMAR, nystagmus, foveal hypoplasia grade 2, and misrouting. Ocular pigmentation for FHONDA and the albinism subtypes is shown in Figure 4.

## Discussion

In this study, we described the genotypic and phenotypic characteristics of the rare FHONDA syndrome and compared it to the phenotype of the most common types of albinism, OCA1, OCA2, and OA1. To date, only 33 patients with the FHONDA syndrome were reported in the literature.^6^^–^^8^^,^^14^^,^^16^^–^^20^

### Genotype

The *SLC38A8* gene consists of 10 exons (all coding), spanning almost 32.4 kb of genomic DNA in the region of 16q23.3. This gene encodes the Solute carrier family 38 member 8 protein, a 46.9 kDa putative sodium-coupled neutral amino acid transporter (SNAT) with 11 transmembrane domains, which consists of 435 amino acids. The protein is expressed in the central nervous system and neuronal retina, predominantly in the inner and outer plexiform layers and photoreceptor layers. *SLC38A8* has a possible broad substrate profile with high preference for transporting glutamate.^8^^,^^26^

Including all patients from the literature, 15 of the 18 identified missense mutations are localized in, or very near, transmembrane regions (see Fig. 1, Supplementary Table S2). Mutations probably affect the transport function of the protein. The p.(Thr87Ile) and p.(Asp283Ala) are both localized on the extracellular side of the channel and cause a change to a hydrophobic amino acid. Toral et al. postulated that p.(Asp283Ala) missense mutation results in a more positive electrostatic potential at the extracellular side of the channel, potentially disrupting the local concentration of sodium and affecting glutamine transport.^17^ However, the exact mechanisms of the pathogenicity of the identified missense mutations are still unknown.

The identified nonsense, splice, deletion, and frameshift mutations are spread over the entire gene. They result in translation of a truncated protein or reduced amount of protein due to nonsense mediated decay, thus, affecting localization and/or transport function of the protein. A *SLC38A8* functional analysis test is needed to determine the pathological effects of the identified mutations.

It is noteworthy that the c.848A > C; p.(Asp283Ala) mutation was detected in all Ashkenazi Jewish patients reported in this study, and 2 Ashkenazi Jewish patients from the study of Toral et al*.*^17^ In total, five patients from three families were homozygous, and one patient of mixed Ashkenazi descent was compound heterozygous. In all Indian Jewish patients from this study, and Indian and Karaite Jewish patients reported in the literature, the mutation c.95T > G; p.(Ile32Ser) was discovered, 17 patients from 8 families were homozygous and one patient was compound heterozygous (see Table 1, Supplementary Table S2). This mutation was not seen in other patients. The six novel *SLC38A8* mutations identified in our study significantly extend the genotypic heterogeneity among patients with FHONDA.

### Phenotype

All 38 different mutations that are discovered until now in the *SLC38A8* gene resulted in similar phenotypes, comprising poor VA, nystagmus, severe foveal hypoplasia (grade 4 in our study, and grade 3 or 4 in previously reported patients), definite chiasmal misrouting, and no signs of any pigmentation defect (skin, hair, iris, or fundus). All newly diagnosed and all but two of the previously reported patients fit this profile.^5^^–^^9^^,^^17^^–^^20^ These two patients seem to express some amount of hypopigmentation. First, patient P4, one of 990 patients from an albinism study by Lasseaux et al. had iris translucency.^14^ Further details of this patient were unavailable, so we were unable to determine if this translucency could have other causes. The other patient from this study that had iris translucency was excluded from the series (personal communication). In addition, one patient described by Kuht et al. had mild iris transillumination, but this patient also had a *TYR* variant which could explain the pigmentation defect of the iris. In three patients, some hypopigmentation of the fundus was described. In all patients, the pigmentation could be considered normal for age, because two patients were only a few months old. The only adult was of white British background and she had no hypopigmentation compared to her family.^16^^,^^19^

It is noteworthy that, even though we detected 6 various novel *SLC38A8* mutations among our patients, this did not change the phenotypic homogeneity. The only variable phenotypic characteristic of FHONDA appears to be anterior segment dysgenesis (ASD), mostly consisting of posterior embryotoxon/Axenfeld's anomaly. This was found in only four patients from three families in this study, and seven patients from three families of previously reported patients (see Table 2, Supplementary Table S3). The genetic changes in four families with posterior embryotoxon were solely detected in these patients, and not in any patients without ASD. However, differences in genotype cannot fully explain the presence or absence of ASD, because mutations in the remaining three families with ASD were also observed in patients without any ASD. This means that posterior embryotoxon/Axenfeld's anomaly is not a frequent abnormality in this disorder, occurring in less than 19% of the patients, which is within the range of prevalence in the normal population (7–-32%).^27^^–^^30^

### FHONDA Versus Albinism

It is interesting that FHONDA appears to have a more narrow phenotypic spectrum compared to albinism, especially with regard to nystagmus, grade of foveal hypoplasia, and chiasmal misrouting. Mutations in several genes can cause different subtypes of albinism, but within one genetic subtype, the phenotypic spectrum is still broad.^10^^,^^11^^,^^13^^,^^14^ A previous study on albinism concluded that absence of photoreceptor specialization (grades 3 and 4 foveal hypoplasia) was associated with worse VA in albinism, with grade 4 associated with the poorest VA.^10^ Photoreceptor differentiation was affected in all patients with FHONDA, which may explain the significantly poorer VA than in albinism. Besides poorer VA and more severe foveal hypoplasia, misrouting was detected with all VEP test stimuli with chiasm coefficients not higher than −0.45. This could be explained by, on average, more crossing of the optic nerve fibers at the chiasm than in albinism, so that noise is not affecting the signal as much (noise causes a shift of the chiasm coefficient towards zero).^23^ This theory is also supported by the findings of Ahmadi et al., who demonstrated that in a patient with FHONDA, all temporal retinal fibers project to the contralateral hemisphere, instead of excessive crossing only in most patients with albinism.^31^

Despite the recently described diagnostic criteria for albinism, the clinical distinction between patients with FHONDA of Caucasian descent and patients with OA1 without evident ocular hypopigmentation might still be difficult. For instance, mild hypopigmentation of the (mid)peripheral fundus can be normal in a lightly pigmented Caucasian family, as is seen in the patient with FHONDA VII-811 and in proband 2 described by Campbell et al*.* In our OA1 cohort, we identified only three patients without ocular hypopigmentation. One patient was mildly affected, in contrast to FHONDA, with better VA, and grade 1 or 2 foveal hypoplasia. In the other two patients, fundus hypopigmentation could be related to an overall light skinned Caucasian phenotype and high myopia, respectively. Of these two patients, one resembled FHONDA closely (VA 0.5 logMAR, nystagmus, foveal hypoplasia grade 3, and misrouting), whereas the other patient did not have misrouting, which has not yet been reported in patients with FHONDA. Therefore, only in 1 of 43 patients with OA1 the differentiation with FHONDA could not be clearly made. To further differentiate the two disorders, in a recent study describing the phenotypic spectrum of albinism, poor visual acuity, grade 4 foveal hypoplasia, and misrouting were always accompanied by iris translucency or other obvious signs of hypopigmentation.^10^ This means that in a severely affected patient but without evident hypopigmentation the possibility of FHONDA is most likely (see Fig. 2). It should be noted, however, that this conclusion is based on a small number of patients with FHONDA. The identification of only 3e patients with mutations in *SLC38A8* in a series of 990 presumed patients with albinism, and only 61 patients with FHONDA reported to date confirm that this is a rare entity.^14^ We hope that this study will raise physicians’ awareness to this uncommon disease and that *SLC38A8* will be added to gene panels for nystagmus.

This study confirms that, in consent with the findings of Poulter et al., FHONDA and albinism appear to be different entities, and the combination of foveal hypoplasia and misrouting of the optic nerve fibers can occur independently of abnormal melanin synthesis. It is therefore plausible that a common pathway exists that causes both foveal hypoplasia and misrouting. The uniformly severe phenotype of FHONDA suggests that SLC38A8 might be located at the end of this pathway, without the possibility of a partial rescue.

Although Ehprin-B1 was previously identified as playing a key role in ipsilateral guidance in species with a small part of the ventral retina projecting ipsilaterally, Hoffmann et al. did not detect an association with visual pathway abnormalities in humans with Ephrin-B1 deficiency.^32^^–^^35^ We hypothesize that other components of the Eph system may play an important role in this pathway. Ephrins are a family of membrane-tethered proteins that serve as ligands of the Eph receptor. Eph/ephrin signaling regulates many developmental processes, including the guidance of axonal growth of cones, and formation of tissue boundaries.^36^^–^^38^

The formation of a foveal avascular zone (FAZ) is necessary for normal foveal development.^39^ Kozulin et al. showed involvement of pigment epithelium derived factor (PEDF) and EphA6 in the definition of the FAZ.^40^^,^^41^ During formation of the FAZ, EphA6 levels start to rise in the macular region and continue to rise after birth while cone differentiation and elongation of the most central cones occurs, a process that appears not to take place in patients with grade four foveal hypoplasia.^39^^–^^41^ During axon guidance, EphA6 expression is highest in the temporal retina and gradually drops around the central retina, implying an important role of EphA6 in retinal ipsilateral axon guidance.^34^^,^^39^ Therefore, EphA6 might be a promising candidate for a common pathway causing foveal hypoplasia and excessive crossing of the optic nerve fibers at the chiasm.

In conclusion, we describe the mutational spectrum, including six novel mutations, and narrow phenotypic spectrum of FHONDA, consisting of poor VA, nystagmus, severe foveal hypoplasia (grade 3 or 4), and chiasmal misrouting. Our study confirms that lack of pigmentation is not essential to cause the combined occurrence of foveal hypoplasia and misrouting. This, in turn, may have important implications for current research on the pathogenesis of these abnormalities and future therapeutic options for alleviation of ocular anomalies leading to vision loss in albinism.^42^^,^^43^

## Supplementary Material

Supplement 1

Supplement 2

Supplement 3
