# Supplementary material for: The Phenotypic and Mutational Spectrum of the FHONDA Syndrome and Oculocutaneous Albinism: Similarities and Differences
Source: Invest Ophthalmol Vis Sci. 2022 Jan 14;63(1):19. doi: 10.1167/iovs.63.1.19 (PMC8762694; doi:10.1167/iovs.63.1.19)
Supplement: Supplement 1 [file iovs-63-1-19_s001.pdf]

Supplemental table S1. Grading scheme for foveal hypoplasia according to Thomas *et al*. [20]

**Grade of foveal hypoplasia**	**Optical coherence tomography findings**
**Grade 0**	Normal foveal structure
**Grade 1**	No extrusion of plexiform layers, a foveal pit is still present, other structures are normal
**Grade 2**	Absence of a foveal pit, underlying foveal structures are normal
**Grade 3**	No foveal pit, no lengthening of the outer segments, normal outer nuclear layer
**Grade 4**	No foveal pit, no lengthening of the outer segments, no widening of the outer nuclear layer
