# Supplementary material for: The Phenotypic and Mutational Spectrum of the FHONDA Syndrome and Oculocutaneous Albinism: Similarities and Differences
Source: Invest Ophthalmol Vis Sci. 2022 Jan 14;63(1):19. doi: 10.1167/iovs.63.1.19 (PMC8762694; doi:10.1167/iovs.63.1.19)
Supplement: Supplement 2 [file iovs-63-1-19_s002.pdf]

Supplemental table S2. Molecular analyses FHONDA patients from literature*

| **Family** | **Descent** | **Mutations SLC38A8 gene** |
|---|---|---|
| Poulter *et al.* F1:IV:1[6,7,14] | *Pakistani* | Homozygous c.707T>A; p.(Val236Asp) |
| Poulter *et al.* F1:IV:3[6,7,14] | *Pakistani* | Homozygous c.707T>A; p.(Val236Asp) |
| Poulter *et al.* F1:IV:4[6,7,14] | *Pakistani* | Homozygous c.707T>A; p.(Val236Asp) |
| Poulter *et al.* F1:IV:6[6,7,14] | *Pakistani* | Homozygous c.707T>A; p.(Val236Asp) |
| Poulter *et al.* F1:IV:7[6,7,14] | *Pakistani* | Homozygous c.707T>A; p.(Val236Asp) |
| Poulter *et al.* F4:II:1[7] | *Pakistani* | Homozygous c.1029del; p.(Leu344Cysfs*7) |
| Poulter *et al.* F5:IV:1[7] | *Turkish* | Homozygous c.101T>G; p.(Met34Arg) |
| Poulter *et al.* F6:IV:7[7,16] | *Indian* | Homozygous c.697G>A; p.(Glu233Lys) |
| Poulter *et al.* F6:VI:1[7,16] | *Indian* | Homozygous c.697G>A; p.(Glu233Lys) |
| Poulter *et al.* F6:VI:2[7,16] | *Indian* | Homozygous c.697G>A; p.(Glu233Lys) |
| Toral *et al*. II:1[15] | *Ashkenazi-Jewish* | Homozygous c.848A>C; p.(Asp283Ala) |
| Toral *et al.* II:2[15] | *Ashkenazi-Jewish* | Homozygous c.848A>C; p.(Asp283Ala) |
| Campbell *et al.* proband 2[17] | *British* | c.534C>G; p.(Ile178Met) and c.(189+1_190-1)_(690+1_691-1)del; p.(?) deletion exon 2 to 5 |
| Weiner *et al.* F1:II:1[18] | *Karait Jewish* | Homozygous c.95T>G; p.(Ile32Ser) |
| Weiner *et al.* F1:II:2[18] | *Karait Jewish* | Homozygous c.95T>G; p.(Ile32Ser) |
| Weiner *et al.* F2:II:3[18] | *Karait Jewish* | Homozygous c.95T>G; p.(Ile32Ser) |
| Weiner *et al.* F3:II:1[18] | *Indian Jewish* | Homozygous c.95T>G; p.(Ile32Ser) |
| Weiner *et al.* F3:II:2[18] | *Indian Jewish* | Homozygous c.95T>G; p.(Ile32Ser) |
| Weiner *et al.* F4:II:1[18] | *Indian Jewish* | Homozygous c.95T>G; p.(Ile32Ser) |
| Weiner *et al* F5:II:1[18] | *Indian Jewish* | c.95T>G; p.(Ile32Ser) and c.490-491del; p.(Leu164Valfs*41) |
| *Lasseaux et al* P2[13] | *Caucasian* | c.527C>G; p.(Thr176Arg) and c.848A>C; p.(Asp283Ala) |
| *Lasseaux et al* P4[13] | *Caucasian* | c.6_9del; p.(Glu2Aspfs*32) and c.923C>G; p.(Thr308Ser) |
| *Kuht et al* F1:II-1[14] | *Korean* | c.692G>A; p.(Cys231Tyr) and c.964C>T; p.(Gln322*) |
| *Kuht et al* F1:II-2[14] | *Korean* | c.692G>A; p.(Cys231Tyr) and c.964C>T; p.(Gln322*) |

| | | |
|---|---|---|
| *Kuht et al* F2:II-1[14] | *Korean* | c.558C>A; p.(Tyr186*) and c.1078_1104del; p.(Ala360_Leu368del) |
| *Kuht et al* F3:II-1[14] | *Korean* | c.995dupG; p.(Trp333Metfs*35) and c.1214+5G>C |
| *Kuht et al* F4:II-1[14] | *Korean* | c.855G>C; p.(Leu285Phe) and c.995dupG; p.(Trp333Metfs*35) |
| *Kuht et al* F5:II-1[14] | *Korean* | c.644G>T[†]; p.(Trp215Leu), c.682G>A[†]; p.(Gly228Arg) and c.695A>G; p.(His232Arg) |
| *Kuht et al* F5:II-2[14] | *Korean* | c.644G>T[†]; p.(Trp215Leu), c.682G>A[†]; p.(Gly228Arg) and c.695A>G; p.(His232Arg) |
| *Kuht et al* F6:II-2[14] | *Korean* | c.954-1G>C and c.995dupG; p.(Trp333Metfs*35) |
| *Kuht et al* F7:II-3[14] | *Turkish* | Homozygous c.101T>G; p.(Met34Arg) |
| *Kuht et al* F8:II-1[14] | *Caucasian* | Homozygous c.632+2T>G |
| *Kuht et al* F9:II-1[14] | *Caucasian* | Exon 1 deletion and c.1126G>A; p.(Gly376Arg) |

*FHONDA patient P1 from Lasseaux *et al* was discarded from their series.[13] [†]These two variants were present in cis configuration[14]
