# Supplementary material for: The Phenotypic and Mutational Spectrum of the FHONDA Syndrome and Oculocutaneous Albinism: Similarities and Differences
Source: Invest Ophthalmol Vis Sci. 2022 Jan 14;63(1):19. doi: 10.1167/iovs.63.1.19 (PMC8762694; doi:10.1167/iovs.63.1.19)
Supplement: Supplement 3 [file iovs-63-1-19_s003.pdf]

Supplemental table S3. Phenotype FHONDA patients from literature*

Family-ID	Refraction RE, LE[†]	VA‡	Nystagmus	Iris translucency	Foveal Hypoplasia[§]	Anterior segment	Hypopigmentation	Misrouting
Poulter *et al.* F1:IV:1[6,7,14]	-0.25/-3.25x175<br>-0.50/-3.00x10	0.8	Yes	No	Grade 4	Axenfeld's anomaly	No	Yes
Poulter *et al*. F1:IV:3[6,7,14]	+6.50/-2.00x172<br>+6.00/-2.00x168	0.8	Yes	No	Grade 4	Posterior embryotoxon	Unknown	Yes
Poulter *et al.* F1:IV:4[6,7,14]	+4.50/-3.00x5<br>+4.25/-3.50x5	1.0	Yes	No	ND	Posterior embryotoxon	No	ND
Poulter *et al*. F1:IV:6[6,7,14]	ND	ND	Yes	No	ND	Posterior embryotoxon	Unknown	ND
Poulter *et al*. F1:IV:7[6,7,14]	+2.50/-2.25x180<br>+3.50/-3.25x180	1.3	Yes	No	ND	Axenfeld's anomaly	Unknown	ND
Poulter *et al.* F4:II:1[7]	ND	ND	Unknown	No	ND	Normal	Unknown	Inconclusive
Poulter *et al.* F5:IV:1[7]	ND	ND	Unknown	No	ND	Normal	Normal	Yes

Poulter *et al*. F6:IV:7[7,16]	+11.00<br>+10.00	1.0	Yes	No	ND	Bilateral micropthalmia and unilateral retinochoroidal coloboma and uveal coloboma	No	ND
Poulter *et al.* F6:VI:1[7,16]	+8.50/-2.00x160<br>+7.50/-1.25x20	0.7	Yes	No	ND	Bilateral micropthalmia	No	ND
Poulter *et al.* F6:VI:2[7,16]	+7.00/-2.75x10<br>+6.50/-2.0x180	0.7	Yes	No	ND	Normal	No	ND
Toral *et al*. II:1[15]	ND	0.6	Yes	No	Grade 4	Normal	No	ND
Toral *et al.* II:2[15]	ND	0.7	Yes	No	Grade 4	Normal	No	ND
Campbell *et al.* proband 2[17]	ND	ND	Yes	No	Grade 4	Posterior embryotoxon	Hypopigmentation in (mid)perifery	Yes
Weiner *et al.* F1:II:1[18]	ND	0.5	Yes	No	ND	Normal	No	ND
Weiner *et al.* F1:II:2[18]	ND	0.5	Yes	No	ND	Normal	No	Yes
Weiner *et al.* F2:II:3[18]	ND	ND	Yes	No	Grade 4	Posterior embryotoxon	No	ND

Weiner *et al.* F3:II:1[18]	ND	ND	Yes	No	ND	Normal	No	ND
Weiner *et al.* F3:II:2[18]	ND	ND	Yes	No	ND	Normal	No	ND
Weiner *et al.* F4:II:1[18]	ND	0.9	Yes	No	ND	Normal	No	ND
Weiner *et al* F5:II:1[18]	ND	ND	Yes	No	ND	Normal	No	ND
Lasseaux *et a* P2[13]	ND	0.5	Yes	No	ND	ND	No	ND
Lasseaux *et al* P4[13]	ND	0.5	Yes	Yes	ND	ND	No	ND
Kuht et al F1:II-1[14]	+2.00/ -3.00 x180 +2.00/-3.00x180	0.5	Yes	No	Grade 3	Normal	No	Yes
Kuht et al F1:II-2[14]	-0.25/-3.00x180 -0.50/-4.00x180	0.7	Yes	No	Grade 3	Normal	No	Yes
Kuht et al F2:II-1[14]	+2.75/-3.50x180 -3.25/-2.75x15	0.3	Yes	No	Grade 3	Normal	No	Yes
Kuht et al F3:II-1[14]	+2.00/-3.00x180 +2.00/-4.00x180	ND	Yes	No	ND	Normal	Hypopigmentation	Yes

Kuht et al F4:II-1[14]	+3.25/-2.00x180 +2.75/-2.50x180	0.4	Yes	No	Grade 3	Normal	No	Yes
Kuht et al F5:II-1[14]	+4.50/-4.00x170 +5.00/-3.00x180	0.3	Yes	No	Grade 3	Normal	No	ND
Kuht et al F5:II-2[14]	+1.75/-3.50x180 +2.00/-3.50x180	0.3	Yes	No	Grade 3	Normal	No	ND
Kuht et al F6:II-2[14]	+2.00/-2.00x180 +3.25/-2.50x10	0.4	Yes	No	Grade 3	Normal	No	Yes
Kuht et al F7:II-3[14]	+1.00/-2.00x180 +1.00/-2.00x180	0.6	Yes	No	Grade 3	Normal	No	Yes
Kuht et al F8:II-1[14]	+3.75/-4.50x180 +4.00/-4.00x180	0.6	Yes	Yes	Grade 4	Normal	No	Yes
Kuht et al F9:II-1[14]	+1.25/-1.25x160 +1.00/-1.00x180	0.6	Yes	No	Grade 4	Normal	No	Yes

ND=not determined. ***** FHONDA patient P1 from Lasseaux *et al* was discarded from their series.[13] †Refraction right eye (RE) and left eye (LE). ‡Visual acuity in logMAR. §According to the grading of Thomas *et al.*[19]
